# Provision of deworming intervention to pregnant women by antenatal services in countries endemic for soil-transmitted helminthiasis

**DOI:** 10.1371/journal.pntd.0007406

**Published:** 2019-05-13

**Authors:** Mathieu Bangert, Pilar Bancalari, Denise Mupfasoni, Alexei Mikhailov, Albis F. Gabrielli, Antonio Montresor

**Affiliations:** Department of Control of Neglected Tropical Diseases, World Health Organization, Geneva, Switzerland; IRNASA, CSIC, SPAIN

## Abstract

**Background:**

The World Health Organization has recently reemphasized the importance of providing preventive chemotherapy to women of reproductive age in countries endemic for soil-transmitted helminthiasis as they are at heightened risk of associated morbidity. The Demographic and Health Surveys (DHS) Program is responsible for collecting and disseminating accurate, nationally representative data on health and population in developing countries. Our study aims to estimate the number of pregnant women at risk of soil-transmitted helminthiasis that self-reported deworming by antenatal services in endemic countries that conducted Demographic and Health Surveys.

**Methodology/Principal findings:**

The number of pregnant women living in endemic countries was extrapolated from the United Nations World Population Prospects 2015. National deworming coverage among pregnant women were extracted from Demographic and Health Surveys and applied to total numbers of pregnant women in the country.

Sub-national DHS with data on self-reported deworming were available from 49 of the 102 endemic countries. In some regions more than 73% of STH endemic countries had a DHS. The DHS report an average deworming coverage of 23% (CI 19–28), ranging from 2% (CI 1–3) to 35% (CI 29–40) in the different regions, meaning more than 16 million pregnant women were dewormed in countries surveyed by DHS. The deworming rates amongst the 43 million pregnant women in STH endemic countries not surveyed by DHS remains unknown.

**Conclusions/Significance:**

These estimates will serve to establish baseline numbers of deworming coverage among pregnant women, monitor progress, and urge endemic countries to continue working toward reducing the burden of soil-transmitted helminthiasis. The DHS program should be extended to STH-endemic countries currently not covering the topic of deworming during pregnancy.

## Author summary

Soil-transmitted helminths are intestinal worms that cause significant suffering among the poorest communities in the world. They are transmitted via contaminated water, food or soil, all of which result from poor sanitation. Children and women of reproductive age are at heightened risk of related morbidities such as malnutrition, cognitive impairment and anaemia. Pregnant women are particularly susceptible to severe maternal and neonatal complications. Deworming drugs are cheap, safe, and effective in reducing morbidity related to soil-transmitted helminthiasis. Large scale drug administration campaigns have distributed donated medicines to children in endemic countries, but women of reproductive age are currently not well covered. Yet, demographic surveys show that they are being treated for soil-transmitted helminthiasis through health care services. This study provides estimates for the number of pregnant women at risk of soil-transmitted helminthiasis being dewormed by antenatal services in endemic countries conducting Demographic Health Surveys. These estimates mark the preliminary reference point for deworming coverage among pregnant women in endemic countries, and will thus prove useful for tracking overall progress in the ongoing effort to eliminate neglected tropical diseases.

## Introduction

Soil-transmitted helminthiasis (STH) are caused by the intestinal worms *Ascaris lumbricoides* (roundworm,) *Trichuris trichiura* (whipworm), *Necator americanus* and *Ancylostoma duodenale* (hookworm). STH are transmitted when individuals come in contact with environment contaminated by faeces containing nematode eggs. The parasites’ eggs or larvae enter the human body via ingestion of infested food, the larvae by skin penetration[1,2].

More than two billion people [1] in 102 endemic countries [3] were considered to be infected with STH in 2015, causing a loss of 39 million of disability-adjusted life years. Most of the disease burden occurs in the tropical and sub-tropical areas of sub-Saharan Africa and South East Asia [4]. STH endemicity is a result of poor sanitary infrastructure and lack of understanding regarding the importance of safe disposal of faeces characteristic of the most impoverished communities of the world [5].

The World Health Organization (WHO) recommends administration of albendazole (400 mg) or mebendazole (500 mg) coupled with hygiene education to reduce STH-related morbidity among high risk populations. Annual PC is recommended in areas where STH prevalence is between 20% and 50%, and biannual PC in areas where STH prevalence exceeds 50%[1]. Completely eliminating STH requires long-term commitment and the allocation of extensive resources toward improving water and sanitation[6].

WHO recognizes three population groups at highest risk of STH-related morbidity: preschool-age children (pre-SAC), school-age children (SAC), and women of reproductive age (WRA)[1]. WRA are especially vulnerable as they are at elevated risk of certain comorbidities such as anaemia (exacerbated particularly by hookworm and whipworm infections)[7]. Drug donations are presently available for the regular PC of pre-SAC and SAC in STH-endemic countries. These countries consistently report the use of donated drugs among risk groups to WHO[8]. In 2015, the implementation of PC averted over 44% of lost disability-adjusted life years due to STH in pre-SAC and SAC [9].

WHO has recently reiterated the importance of WRA as a target population for the scaling up of PC in the Bellagio Declaration[7] as STH among pregnant women can produce or seriously aggravate maternal and neonatal complications[4]. Yet, anthelminthic drug donations are currently not available for WRA and countries are not reporting coverage, making the estimation of PC provided to this group difficult to assess.

In this study, we quantified the number of pregnant women being dewormed by health services in countries considered endemic for STH (>20% prevalence) [3,8] that conducted recent Demographic and Health Surveys (DHS). The estimates offered by this study will provide useful information for the scaling up of PC programmes among WRA, however it is important that control programme managers collect further coverage and context specific epidemiological data to improve these estimates.

## Methods

### United nations world population prospects

The United Nations World Population Prospects 2015 consists of population estimates and projections executed by the Population Division of the Department of Economic and Social Affairs of the United Nations Secretariat. It includes results from national population censuses and demographic and health surveys, outlining key indicators by region, sub-region, country, and development group. The United Nations World Population Prospects 2015 was used to estimate the total number of pregnant women in STH-endemic countries[10].

### Demographic and Health Surveys

DHS are standardized nationally-representative household surveys implemented over a span of 18–20 months about every five years. The surveys are implemented by ICF International and funded by the United States Agency for International Development (USAID) with contributions from other donors such as UNICEF, UNFPA, WHO, and UNAIDS. The surveys consist of four types of questionnaires (Household, Woman’s, Man’s, and Biomarker) that collect data on demographic, environmental, socio-economic, and health-related characteristics. One of the aim of DHS is to evaluate the performances of health services; in this context DHS asked to women in the sample with a live birth in the past five years, if the “took intestinal parasite drug” during the pregnancy of their last birth [11]. All DHS survey data were extracted and analysed using R statistical program and the rDHS and survey packages. Permission for sub-national DHS data between 2000 and 2017 in STH-endemic countries was first sought, downloaded through the DHS API, and national means and confidence intervals of coverages of deworming among pregnant women by antenatal services summarised using sample weights.

### Total pregnant women

The number of live births obtained from the United Nations World Population Prospects 2015 was assumed to be equal to the total number of pregnant women in each country. The 2015 report was used, as opposed to a more recent version, to maintain consistency with previously estimated deworming coverage among not pregnant WRA.

### National, regional, and global treatment coverage among pregnant women

Deworming coverage measured by DHS were assumed to be representative of the national situation. For DHS reports reporting deworming, the proportion of women dewormed during their last pregnancy was applied to number of pregnant women in each country. Total pregnant women and pregnant women dewormed in each of the endemic countries were aggregated by WHO region and globally.

## Results

### DHS reports

**Fig 1:**
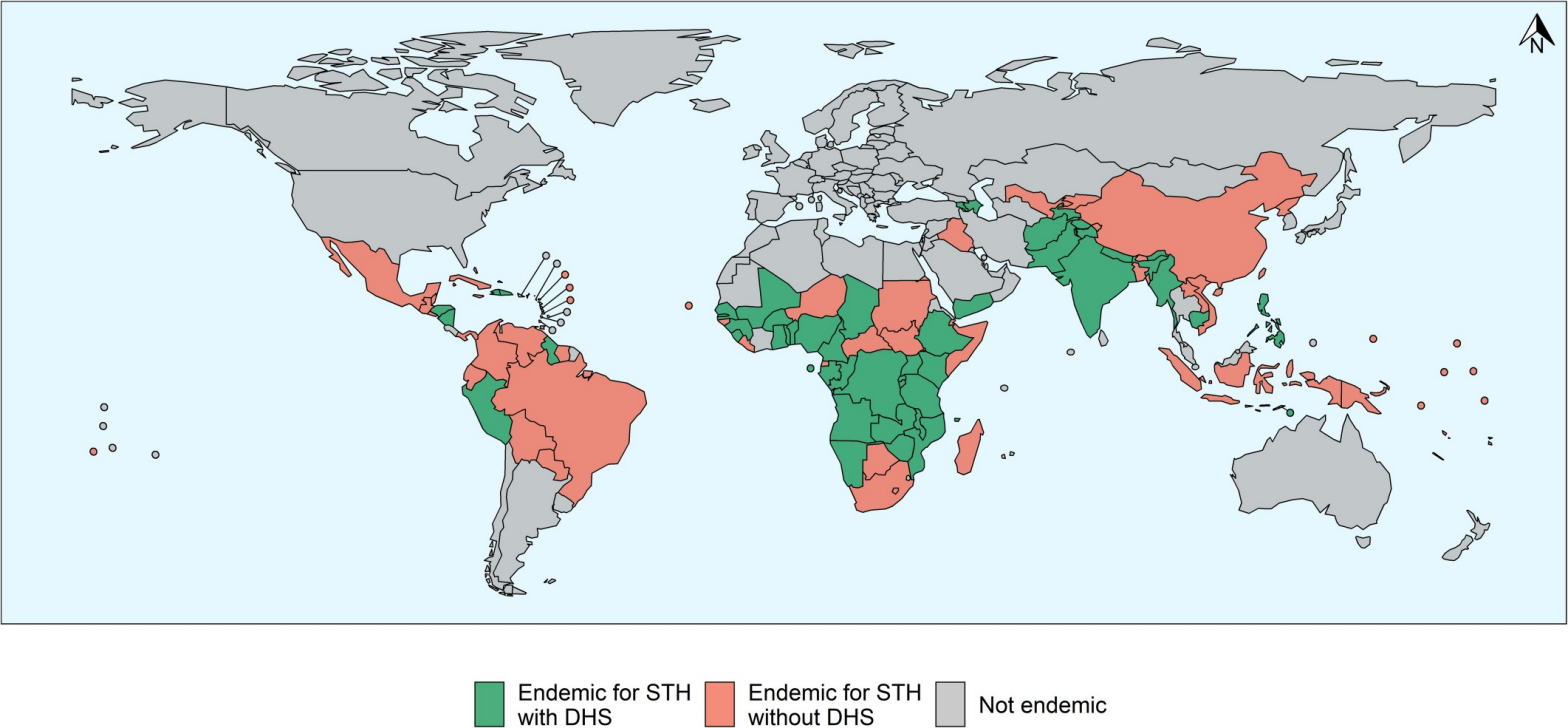
Map showing STH endemic countries, highlighting for which countries DHS data on deworming were available for this study. GIS data by WHO, under ODbL.

Out of 102 STH-endemic countries, DHS reports for 49 countries were available, as depicted in Fig 1. 53 countries did not have DHS reports or were excluded from the analysis as they were based on surveys conducted before 2000. Overall, DHS was available for 48% of all endemic countries (Table 1). Out of the 1.24 million household interviews conducted, 85% were in the South East Asian (636,551) and African (425,650) regions.

**Table 1:** Number of countries endemic for soil-transmitted helminthiasis, number of countries with Demographic and Health Survey reports (DHS) and data on deworming and number of households interviewed as part of the DHS.

WHO region	Number of STH endemic countries	Number of countries for which DHS data on deworming during pregnancy available	Number of households interviewed by DHS
Africa	42	31	425,650
Americas	25	6	79,935
Eastern Mediterranean	7	3	54,689
Europe	5	3	21,505
South East Asia	8	4	636,551
Western Pacific	15	2	30,629
**Total**	102	49	1,248,959

45 out of 49 DHS Reports were based on surveys conducted after 2010, with the majority of surveys being from 2011–2015. Only 6 DHS reports were based on surveys conducted before 2011. The proportion of DHS reports which include the percentage of pregnant WRA dewormed by antenatal services increased from 0% between 2000 and 2005 to 92% after 2016 (see S1 Fig).

### Estimated deworming coverage among pregnant women

Among the 49 countries investigated with DHS, it was estimated that out of 68 million pregnant women at risk of STH, over 16 million received deworming medication during their last pregnancy (Table 2). The kind of anthelminthic drug given was not specified in the DHS reports.

**Table 2:** Estimated number of pregnant women in countries, and estimated number of pregnant women dewormed and not dewormed as derived from Demographic and Health Surveys (DHS).

WHO region	Number of countries	Estimated number of pregnant women^§^ (in 1000s)	Number of pregnant women estimated to be dewormed (in 1000s; CI)	Number of pregnant women estimated not to be dewormed (in 1000s; CI)	Percentage of pregnant women estimated to be dewormed (%; CI)
Africa	31	30,759	10,637 (9,051–12,281)	20,122 (18,478–21,707)	35 (29–40)
Americas	6	1,398	136 (112–165)	1,262 (1,233–1,285)	10 (8–12)
Eastern Mediterranean	3	7,387	154 (116–202)	7,233 (7,185–7,270)	2 (2–3)
Europe	3	488	10 (6–17)	478 (471–481)	2 (1–3)
South East Asia	4	27,358	5,106 (3,949–6,544)	22,252 (20,814–23,408)	19 (14–24)
Western Pacific	2	2,719	361 (308–427)	2,358 (2,292–2,410)	13 (11–16)
**Total**	**49**	**70,109**	**16,404 (13,545–19,636)**	**53,705 (50,473–56,563)**	**23 (19–28)**
Total; not captured by DHS^#^	53	43,320	NA	NA	NA

Notes

# = Countries that are endemic for STH but for which no DHS data on deworming was available at sub-national level

§ = number of live births obtained from the United Nations World Population Prospects 2015

The average coverage of deworming was estimated at 23% (CI 19–28) among the countries surveyed, and varied between 0.6% (CI 0.3–1.2) and 83% (CI 75–89).

Overall, the 49 countries with DHS reports accounted for 62% of total pregnant women in all STH-endemic countries. DHS represented deworming coverage during pregnancy in the African region the best, with 31 out of 42 STH endemic countries surveyed. In contrast, the Western Pacific Region was least represented by DHS, with only 2 out 15 STH endemic countries surveyed for deworming coverage during pregnancy. There was no DHS report available for China, which accounts for 77.8% of pregnant women at risk of STH in the Western Pacific region and 14.6% of pregnant WRA in all endemic countries. Consequently, the Western Pacific region had the lowest percentage of pregnant women represented by available DHS (Table 2).

Table 2 describes the regional distribution of total pregnant women at risk of STH as well as the estimated number of pregnant women dewormed in all STH-endemic countries.

## Discussion

In this study we estimate that in 49 STH endemic countries where DHS was recently conducted over 16 million have received deworming. More than 1.2 million households were interviewed and we consider the estimated deworming coverage representative of the performances of health services in those countries.

At the moment countries are not requested to report coverage data on WRA because this risk group is not among the ones targeted by the NTD road map. Our data shows an overall positive trend in the number of STH-endemic countries reporting deworming of pregnant women in the absence of global monitoring, the initial spike in this trend coinciding with the first Global Partner’s Meeting on Neglected Tropical Diseases organized in 2007 [12]. Deworming initiatives for pregnant women are likely to continue growing as countries scale up their STH control programs. These estimates constitute a valuable step toward the coverage of this group at risk as they represent baseline figures of deworming coverage among pregnant women in multiple endemic countries.

Although we do not have access to more recent DHS information on countries that conducted surveys between 2000 and 2005, it is probable that those countries have since initiated deworming activity for pregnant women. Additionally, we recognize that countries that do not report deworming as a component of antenatal services may be providing anthelminthic drugs to pregnant women through other platforms, or women are procuring deworming tablets in local pharmacies or markets. The coverage of deworming among pregnant women in this study could therefore be underestimated.

It is important to note that we found DHS for 73% of the STH-endemic countries in Africa, and for 50% of the STH-endemic countries in South East Asia, both accounting for the vast majority of pregnant women at risk of STH worldwide. As these two WHO regions comprise the highest concentration of STH, we consider that the data collected is particularly representative of those countries most in need of targeted PC. There was, however, no data on deworming during pregnancy from 53 STH endemic countries, meaning an evidence gap still remains in these countries and should be addressed, particularly in the American and Western Pacific regions, to better understand global deworming coverage in pregnant women. In addition to expanding the geographic scope of DHS, the questionnaire could be adapted in future surveys to elicit better quality data on deworming in WRA by, for example, by limiting the question on use of deworming to the latest pregnancy.

A limitation of this study is the assumption that the number of pregnant women in a country is equal to the number of live births, as it omits all pregnancies that resulted in abortions, miscarriages, and stillbirths. On the other hand, multiple pregnancies (i.e. those giving birth to twins) may lead to overestimation of total number of pregnant women, granted an overall underestimation is more likely.

In our opinion, antenatal services represent a relevant potential channel for systemic PC delivery considering that countries such as Congo have achieved 85.8% deworming coverage of pregnant women solely through antenatal services.

Targeted PC can significantly reduce the burden of STH among WRA, improving maternal and child health outcomes and spurring productivity[13]. Considering the prospective impact of STH elimination and the relatively low implementation costs, PC among WRA should be at the forefront of public health operations in all endemic countries. We urge countries that have already integrated deworming into health programming to continue scaling up the provision of anthelminthic treatment among WRA. The estimates provided by this study will aid strategic efforts by informing the expansion of PC among WRA in endemic countries and promoting the definitive goal of eliminating STH globally.

## Supporting information

S1 Fig Percentage of Demographic and Health Surveys that report deworming rate among pregnant women from 2000–2017, by five-year interval in which they were conducted. (TIF)

## References

[1] World Health Organization. Helminth control in school-age children: a guide for managers of control programmes. 2nd ed Geneva: World Health Organization; 2011.

[2] Gabrielli AF, Montresor A, Savioli L. Soil-Transmitted Helminthiasis In: Bruschi F, editor. Helminth Infections and their Impact on Global Public Health. Vienna: Springer Vienna; 2014 pp. 275–297. 10.1007/978-3-7091-1782-8_9

[3] Schistosomiasis and soil-transmitted helminthiases: number of people treated in 2015 [Internet]. Geneva: World Health Organization; 2016 Dec pp. 585–600. Report No.: 49/50. Available: http://apps.who.int/iris/bitstream/handle/10665/251908/WER9149_50.pdf

[4] Savioli L, Albonico M, Engels D, Montresor A. Progress in the prevention and control of schistosomiasis and soil-transmitted helminthiasis. Parasitology International. 2004;53: 103–113. 10.1016/j.parint.2004.01.001 15081942

[5] Tchuem Tchuenté LA. Control of soil-transmitted helminths in sub-Saharan Africa: Diagnosis, drug efficacy concerns and challenges. Acta Tropica. 2011;120: S4–S11. 10.1016/j.actatropica.2010.07.001 20654570

[6] Larocque R, Casapia M, Gotuzzo E, Gyorkos TW. Relationship between intensity of soil-transmitted helminth infections and anemia during pregnancy. Am J Trop Med Hyg. 2005;73: 783–789. 16222026

[7] World Health Organization. Reaching girls and women of reproductive age with deworming [Internet]. Bellagio, Italy: Rockefeller Foundation Bellagio Center; 2017 Jun. Report No.: WHO/CDS/NTD/PCT/2018.01. Available: http://www.who.int/iris/handle/10665/259962

[8] Mupfasoni D, Mikhailov A, Mbabazi P, King J, Gyorkos TW, Montresor A. Estimation of the number of women of reproductive age in need of preventive chemotherapy for soil-transmitted helminth infections. Knopp S, editor. PLOS Neglected Tropical Diseases. 2018;12: e0006269 10.1371/journal.pntd.0006269 29432423 PMC5825157

[9] Montresor A, Trouleau W, Mupfasoni D, Bangert M, Joseph SA, Mikhailov A, et al Preventive chemotherapy to control soil-transmitted helminthiasis averted more than 500 000 DALYs in 2015. Transactions of The Royal Society of Tropical Medicine and Hygiene. 2017;111: 457–463. 10.1093/trstmh/trx082 29346640 PMC5808863

[10] United Nations, Department of Economic and Social Affairs, Population Division. World population prospects: the 2015 revision [Internet]. 2015 Available: https://esa.un.org/unpd/wpp/publications/files/key_findings_wpp_2015.pdf

[11] ICF. Demographic and Health Surveys [Internet]. Rockville, Maryland: USAID; 2000 2017 Available: http://www.dhsprogram.com.

[12] Report of the Global Partners’ Meeting on Neglected Tropical Diseases [Internet]. Geneva, Switzerland: World Health Organization; 2007 4 Available: http://apps.who.int/iris/bitstream/handle/10665/69740/WHO_CDS_NTD_2007.4_eng.pdf

[13] Bangert M, Molyneux DH, Lindsay SW, Fitzpatrick C, Engels D. The cross-cutting contribution of the end of neglected tropical diseases to the sustainable development goals. Infectious Diseases of Poverty. 2017;6 10.1186/s40249-017-0288-0 28372566 PMC5379574

