# Supplementary figures and images for: Provision of deworming intervention to pregnant women by antenatal services in countries endemic for soil-transmitted helminthiasis
Source: PLoS Negl Trop Dis. 2019 May 13;13(5):e0007406. doi: 10.1371/journal.pntd.0007406 (PMC6532928; doi:10.1371/journal.pntd.0007406)

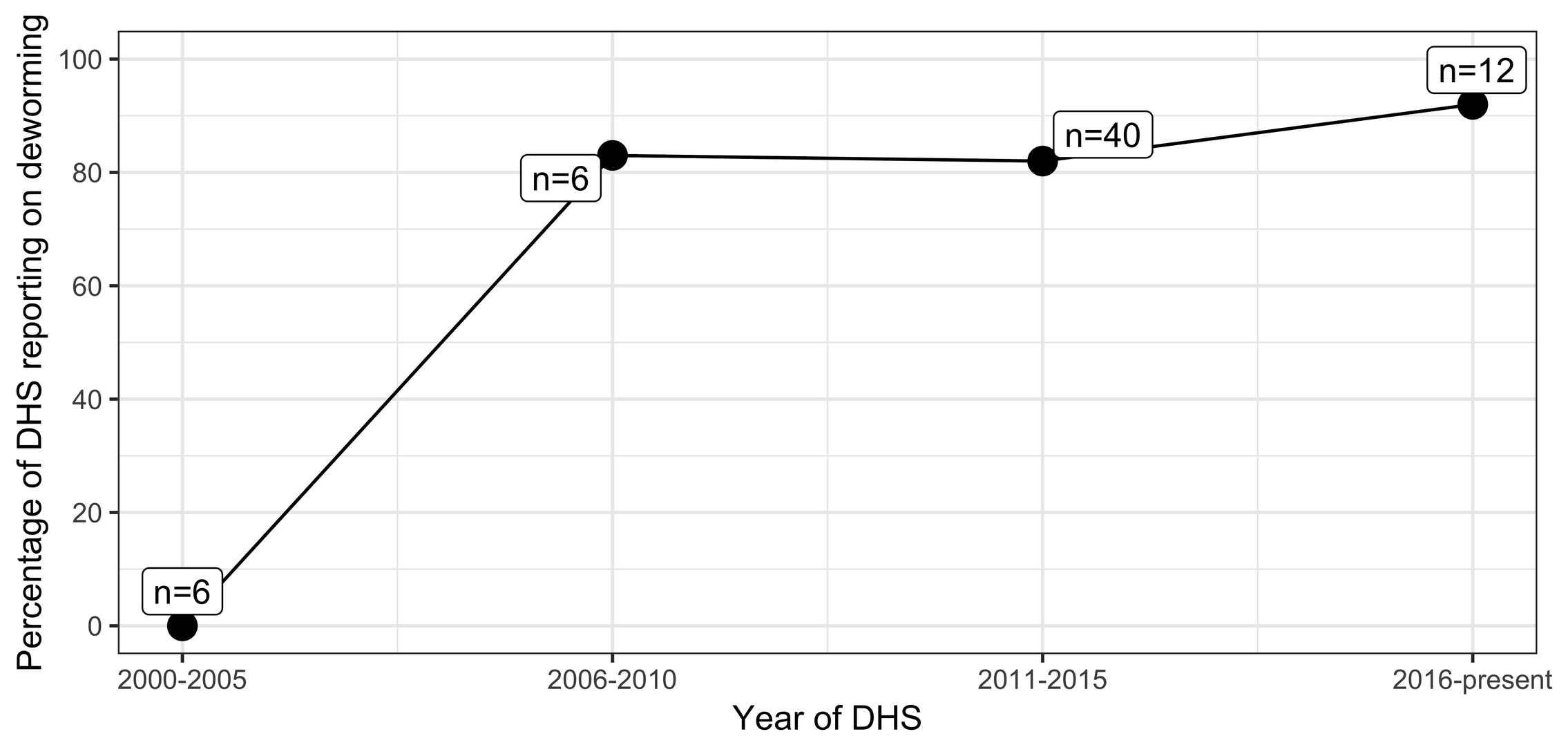

Supplement: S1 Fig — (TIF) [file pntd.0007406.s001.tif]
